# Special Issue “The Role of Toll-like Receptors (TLRs) in Infection and Inflammation: Third Edition”

**DOI:** 10.3390/ijms27198847

**Published:** 2026-10-03

**Authors:** Ralf Kircheis, Oliver Planz

**Affiliations:** 1 Advanced Medical Solutions Saal GmbH, 93342 Saal an der Donau, Germany; 2 Institute of Cell Biology and Immunology, Eberhard Karls University Tuebingen, 72076 Tuebingen, Germany; oliver.planz@uni-tuebingen.de

## 1. Introduction

Toll-like receptors (TLRs) are central players of the host innate immune system, functioning as germline-encoded pattern recognition receptors (PRRs) that monitor extracellular and endosomal environments. Classically defined by their ability to recognize conserved microbial motifs, i.e., pathogen-associated molecular patterns (PAMPs), as well as endogenous danger signals (damage-associated molecular patterns, or DAMPs), TLRs initiate signaling cascades that shape both immediate host defense and downstream adaptive immunity [1,2,3,4,5,6,7,8,9,10,11,12,13,14,15]. Upon ligand binding, these transmembrane proteins undergo conformational rearrangements and specific homo-/heterodimerization events. Ligand-triggered dimerization of TLR brings their intracellular Toll/Interleukin-1 receptor (TIR) domains into proximity, enabling the recruitment of key cytosolic adaptors such as MyD88 and TRIF [16,17,18,19,20,21].

**Figure 1:**
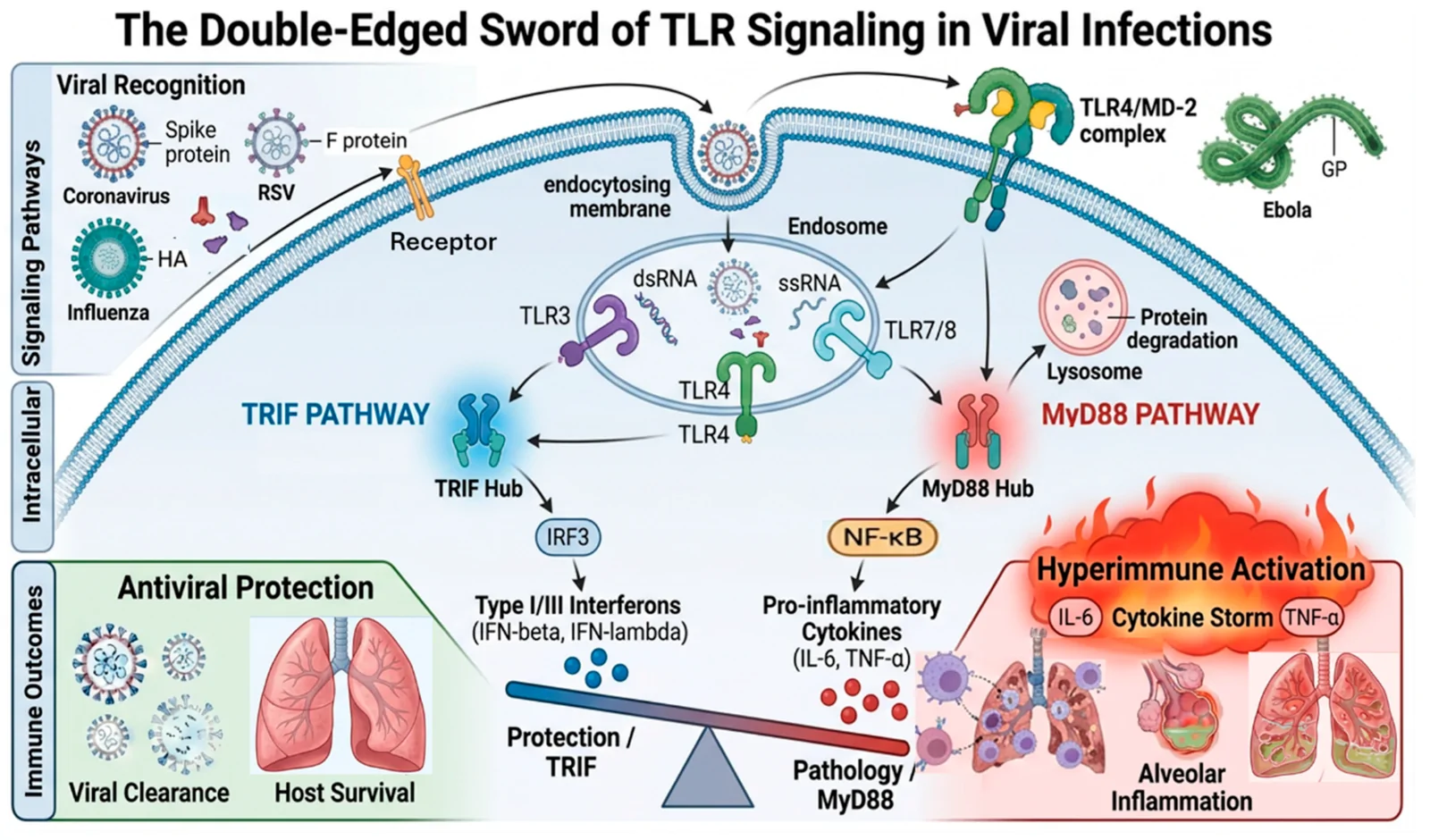
Double-edged sword of TLR signaling in viral infections. Highly pathogenic viruses such as various coronaviruses, influenza virus, RSV, and Ebola virus bind to their host cell receptors followed by uptake into the cells via membrane fusion or via the endosomal pathway. Viral components act as pathogen-associated molecular patterns recognized by a variety of pattern recognition receptors (PRRs), such as Toll-like receptors (TLRs). Signaling of surface-exposed TLRs (e.g., TLR4, TLR2 (in complex with TLR1 or TLR6)) or TLRs in the endosomal compartments (e.g., TLR3, TLR4, TLR7/8) is triggered by ligand (PAMP)-induced homo-/or heterodimerization of the extracellular/luminal part of TLRs, bringing their intracellular Toll/IL-1 Receptor (TIR) domains into juxtaposition, which triggers the recruitment of cytosolic adaptor complexes and associated signaling pathways, such as MyD88/NF-κB or TRIF/IRF3, and resulting in pro-inflammatory cytokine (IL-1, IL-6, TNF-α) or interferon release. Depending on the amplitude and the balance of MyD88/NF-κB vs. TRIF/IFN, activation of TLRs can result in antiviral immune protection or hyperactivation of the innate immune system, cytokine storm, and tissue damage. The provided figure is based on the content from [39,53].

While tightly regulated TLR signaling is essential for pathogen elimination and maintaining tissue homeostasis, its unrestrained or aberrant hyperactivation can act as a double-edged sword. Uncontrolled signaling frequently culminates in persistent low-grade inflammation, or catastrophic systemic “cytokine storms,” or destructive tissue pathologies [22,23,24,25,26,27,28,29,30,31,32,33,34,35], characteristic in particular also for acute infection with highly pathogenic bacteria or viruses [36,37,38,39,40,41,42,43,44,45,46,47,48,49,50,51,52] (Figure 1).

The present Special Issue collection brings together ten research articles that are illustrating the multifaceted roles of TLRs across a broad spectrum of physiological and pathological conditions. By bridging computational structural biology, experimental animal models, and clinical studies, these contributions may contribute to our understanding of TLR networks and highlight innovative therapeutic approaches targeting these central innate immune hubs.

**Figure 2:**
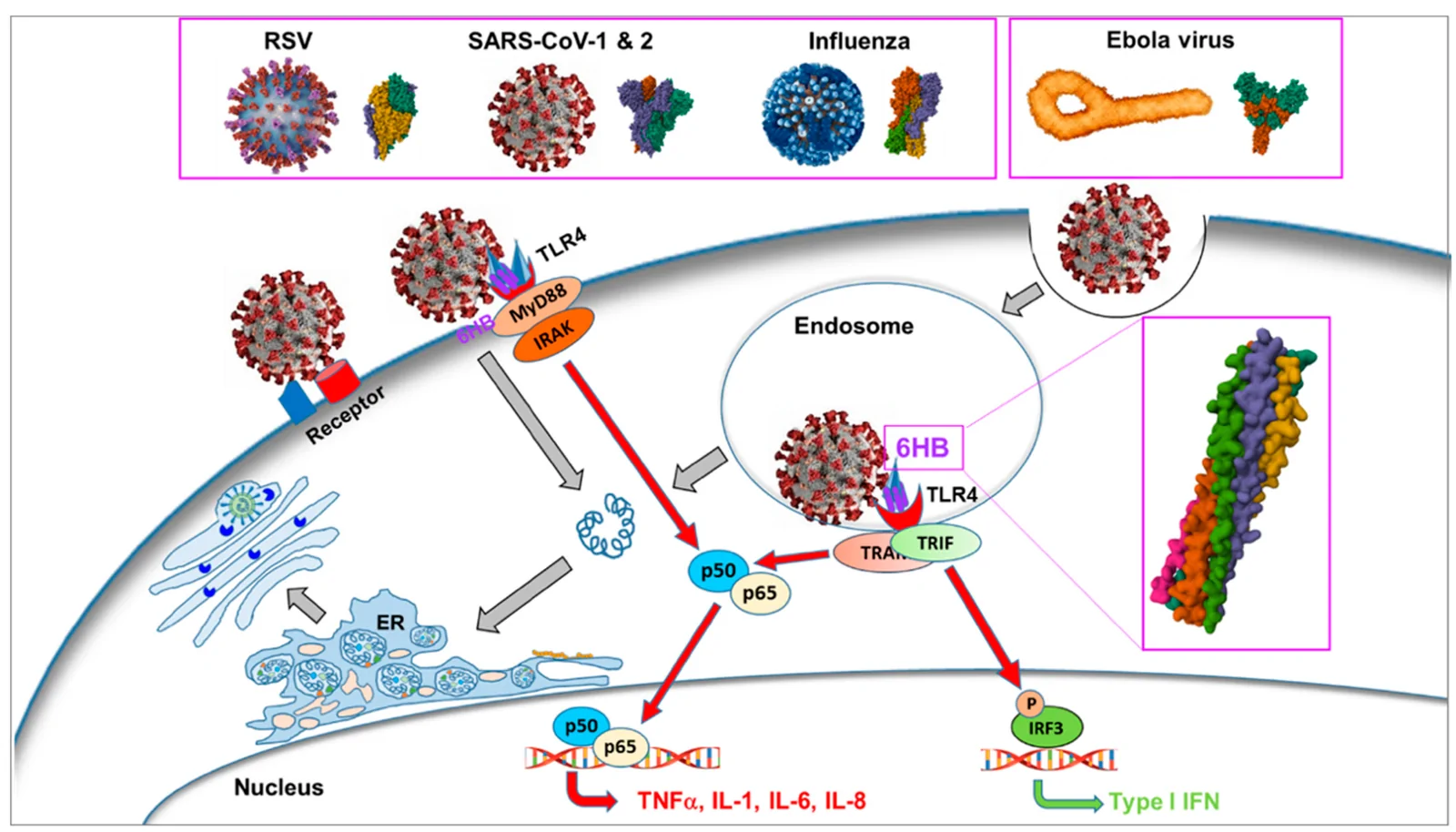
Interconnection of the replication cycle for multiple enveloped viruses such as SARS-CoV, SARS-CoV-2, MERS-CoV, influenza A, B and C, RSV, and Ebola virus at the host membrane/endosome fusion step with TLR4 activation. Viral fusion with host membrane and/or endosomal membrane will lead to formation/exposure of conserved coiled-coil fusion core structures, such as six-helix bundles (6HB), which can trigger dimerization of two TLR4/MD-2 heterodimers, as illustrated for the SARS-CoV-2. SARS-CoV-2 binds via the spike protein to its primary host cell receptor, i.e., ACE2, followed by fusion with the host cell membrane. Viral RNA transcription and translation of viral proteins occur in double-membrane vesicles (DMVs) or endosomal reticulum (ER). The newly assembled virus particles leave the cells via the Golgi apparatus where the spike protein undergoes proteolytic cleavage. This process occurs preferably in virus producer cells with high TMPRSS2 expression, such as alveolar cells (left side). Alternatively, the virus can be taken up into endosomes, predominantly in cathepsin L-rich cells, such as innate immune cells and endothelial cells (right side). Several of the enveloped virus (SARS-CoV, SARS-CoV-2, MERS-CoV, influenza A, B, C) are known to utilize both host cell fusion pathways (i.e., host membrane and endosomal membrane), whereas other prefer entry via host membrane (RSV) or exclusively use endosomal pathway (Ebola virus). Both pathways, however, can result in TLR4 dimerization, leading to signaling towards the activation of the MyD88/NF-κB (p50/p65) pathway and/or TRIF/interferon pathways. The figure is based on the content from [53].

Leading the present collection into a broader biological concept, the first article by Kircheis [53] presents a novel computationally derived hypothesis for a molecular mechanism broadly shared by diverse enveloped viruses which triggers the cytokine storm and hyperactivation of the innate immune system frequently found during acute stages of infection by highly pathogenic viruses. While TLR4 is classically recognized as the sensor for bacterial lipopolysaccharide (LPS), it has been increasingly implicated also in viral hyperinflammation. The in silico molecular docking study reported in this collection indicates that structurally conserved, coiled-coil structures such as post-fusion six-helix bundles (6HB) from highly pathogenic enveloped viruses, including SARS-CoV-2, MERS-CoV, influenza A/B/C, and respiratory syncytial virus (RSV), may engage directly and induce the dimerization of human TLR4/MD-2 heterodimers. This binding, driven by exposed hydrophobic coiled-coil domains and surface-exposed charged residues, bypasses classical lipopolysaccharide (LPS) pocket-binding mechanisms, operating via broader surface binding stabilization. In comparison, TLR4/MD-2 heterodimerization induced by Ebola virus fusion protein GP2 involves both, i.e., interaction by coiled-coil segments with TLR4 and an ideal fit of the extended fusion peptide of GP2 into the groove between TLR4 and MD-2, correlating with a particularly high binding affinity to TLR4/MD-2 (Figure 2). Interestingly, docking studies calculated a significantly decreased TLR4/MD-2 dimerization potency for spike protein variants of SARS-CoV-2 with the Omicron-typical amino acid substitution N969K compared to wild-type, which correlates with the well-known lower pathogenicity of Omicron strains compared to wild-type.

Importantly, this study indicates interconnection points between the replication cycles of enveloped viruses and TLR4 activation, i.e., innate immune system activation, particularly during the fusion steps of the viral envelope with the host membrane or endosome membrane. This interconnection can be very stringent, as in the case of the Ebola virus where viral envelope fusion occurs only at low pH, i.e., only in the endosomes. In contrast, for other viruses, such as, e.g., SARS-CoV-2, the replication and TLR activation can be dissociated to some degree due to fact that reproductive viral replication is mostly occurring in, e.g., ACE2/TMPRSS2 expressing cells, such as alveolar lung cells, which express no or only low amounts of TLRs on their surface. In contrast, TLR4 activation occurs mostly in innate immune cells and endothelial cells expressing high numbers of TLR4/MD-2 but lack ACE2/TMPRSS2 expression, but finally infection in these cells ends up as non-productive. Overall, the study indicates that structurally conserved—and, during viral fusion, exposed—hydrophobic coiled-coil domains and surface-exposed charged residues may directly engage and promote the dimerization of two TLR4/MD-2 heterodimers as one mechanism that seems to be shared by a broad range of enveloped viruses.

The next two investigations are related to the differential effect of TLR signaling in response to viral infections, with focus on other TLRs, such as TLR7 and TLR2. 

Miles et al. reported a complex temporal role for endosomal TLR7 during influenza A virus (IAV) infection using C57BL/6 wild-type and knockout (KO) models. During peak acute viral replication (day 7 post-infection), wild-type mice exhibited severe lung inflammation, characterized by extensive tissue damage, neutrophil infiltration, and a profound Th1/Th17 cytokine storm driven by TLR7-mediated viral RNA sensing. TLR7 knockout protected against this acute lung tissue damage and stiffness. However, a striking kinetic shift occurred during the disease resolution phase (day 14). TLR7 KO mice failed to resolve airway hyperresponsiveness, developing chronic large airway resistance associated with a high influx of eosinophils and CD8+ cytotoxic T cells. This phenotype was traced to enhanced Th2 signaling (elevated IL-5) and impaired regulatory T cell surveillance in the case of TLR7 knockout. The findings suggest that TLR7 plays temporally distinct roles during influenza infection, calling for a stage-dependent therapeutic approach: deploying TLR7 antagonists exclusively during peak acute infection while avoiding chronic suppression to prevent long-term hyperreactive remodeling [54].

In the next study, the genetic variants rs3804099 and rs3804100 in the TLR2 gene and their impact on severe COVID-19 were studied. Investigating genetic polymorphisms within host defense systems, Flores-González et al. analyzed a clinical cohort of 1018 patients suffering from severe COVID-19 and acute respiratory distress syndrome (ARDS). They specifically interrogate two synonymous single-nucleotide polymorphisms (SNPs) within the TLR2 gene, rs3804099 and rs3804100, which have been shown to influence mRNA splicing efficiency. Although case–control comparisons showed that the C allele in either variant was not directly associated with an increased risk of severe ARDS risk, carriers of the C allele (rs3804099 or rs3804100) exhibited an altered TLR-2 mediated cellular response to the SARS-CoV-2 spike protein. When peripheral blood mononuclear cells (PBMCs) were challenged with the SARS-CoV-2 spike protein, wild-type (TT) individuals responded appropriately by downregulating surface TLR-2 levels on non-switched memory B cells and cytotoxic T cells, a marker of physiological receptor internalization. Conversely, C allele carriers failed to execute this internalization, resulting in persistent surface expression accompanied by increased secretion of IL-6, TNF-α, and IL-10 in an TLR2-independent pathway [55].

The delicate balance between detrimental and protective effects of TLR activation is further illustrated by use of a selective TLR2/6 agonist, highlighting how controlled modulation of this pathway can be exploited for diverse therapeutic applications. In this context, Dronamraju et al. investigated the radioprotective potential of the synthetic TLR2/6 agonist fibroblast-stimulating lipopeptide 1 (FSL-1). Total body irradiation (TBI), as encountered following nuclear accidents or radiological incidents, causes severe hematopoietic injury that frequently progresses to sepsis and multi-organ failure. In a murine model, male and female C57BL/6 mice were exposed to a highly damaging mixed radiation field (65% neutrons, 35% gamma radiation) generated by a TRIGA nuclear reactor. Notably, a single dose of FSL-1 administered 12 h before irradiation significantly improved survival while preserving bone marrow architecture and megakaryocyte cellularity. Mechanistically, FSL-1 pre-treatment induced controlled activation of the MyD88–NF-κB signaling pathway before radiation exposure, resulting in a transient increase in hematopoietic cytokines, including IL-6, MIP-1α, and KC. This response protected femoral short-term hematopoietic stem cell (HSC) pools and accelerated the recovery of megakaryocytes, leukocytes, and platelets. 

**Figure 3:**
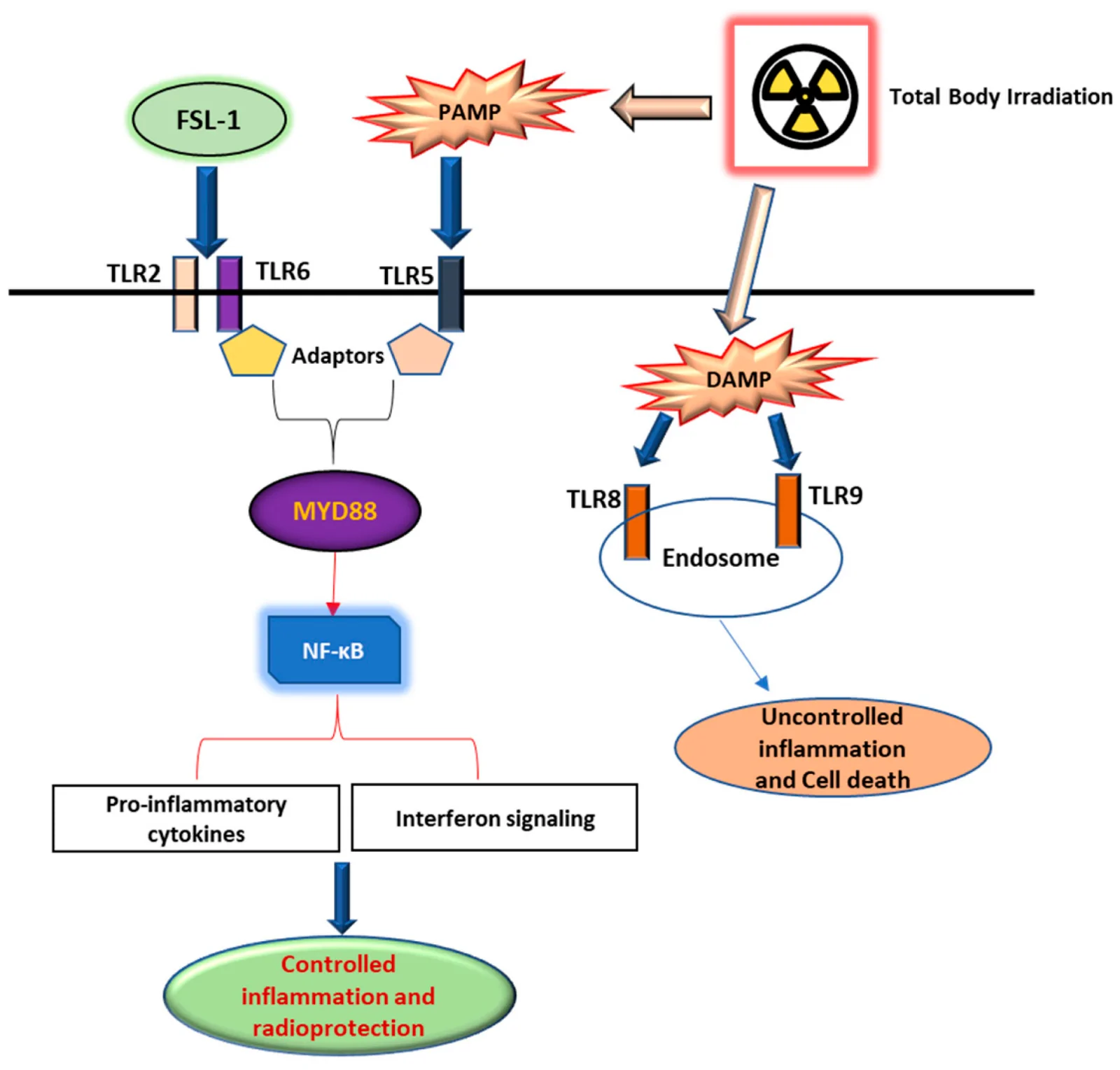
FSL-1 protects against radiation-induced damage through the TLR signaling pathway. FSL-1 given prior to radiation exposure activates the TLR2/6 receptor pathway through Myd88-dependent signaling, thereby enabling controlled pro-inflammatory cytokines and interferon signaling mediated by NF-κB. Conversely, PAMP and DAMP, released extracellularly and intracellularly, activate alternate pathways of inflammatory signaling, regulating cell death and fibrosis. This figure is derived from [56].

By priming the innate immune system in a controlled manner, FSL-1 prevented the profound post-irradiation collapse of hematopoiesis and TLR signaling that otherwise predisposes to opportunistic sepsis and fatal multi-organ failure [56] (Figure 3).

Another approach illustrating the power of fine-tuning of downstream intracellular signaling following TLR4 activation is shown in the next study. Zhang et al. identified DB03476, a novel small-molecule TLR4 inhibitor, through structure-based DrugBank screening targeting the conserved intracellular Toll/IL-1 receptor (TIR) domain. While traditional extracellular domain inhibitors struggle with sensitivity to endogenous damage-associated molecular patterns (DAMPs) like HMGB1, targeting the intracellular convergence point allowed for selective suppression of the Tlr4/MyD88/NF-κB pathway. In a murine ischemia–reperfusion model, DB03476 significantly reduced macrophage infiltration, cellular apoptosis, and the transcription of downstream inflammatory cytokines. Crucially, sequence alignment demonstrated high structural conservation with human TLR4. Using human-induced pluripotent stem cell-derived kidney organoids exposed to macrophage-derived inflammatory cascades, the authors confirmed that DB03476 rescues human renal structures from inflammatory cell death, providing an exciting blueprint for the clinical mitigation of Acute Kidney Injury (AKI) [57].

The next study uncovers an unexpected transcriptional link connecting cytoplasmic nucleic acid sensing to cell-surface bacterial sensing in autoimmune pathology. Di Rosa et al. elucidated a potential mechanism by which the B cell adapter for PI3K (BCAP) modulates systemic lupus erythematosus (SLE) pathophysiology. Evaluating peripheral blood RNA from pediatric SLE cohorts, the authors demonstrated a direct correlation between PIK3AP1 (BCAP) expression and standard type I interferon (IFN-I) signature scores. Cellular tracking revealed that BCAP is directly transcribed as an Interferon-Stimulated Gene (ISG) downstream of the cGAS-STING-TBK1 pathway. Strikingly, functional assays utilizing CRISPR/Cas9-generated BCAP myeloid cells demonstrated that when cells are primed by intracellular nucleic acids, the subsequent upregulation of BCAP acts as a molecular switch. Upon cell-surface exposure to bacterial lipopolysaccharide (LPS), BCAP reroutes Toll-like receptor 4 (TLR4) signal transduction, selectively prioritizing massive IFN-I production over standard pro-inflammatory cytokines like TNF-α. These findings identify BCAP as a potential molecular switch involved in an amplification loop that tips the balance toward IFN-driven immunopathology. Taken together, this study indicates positive feedback loops by which secondary bacterial infections may drive clinical flares in lupus patients by exploiting an innate priming network [58].

The next study tracks TLR4, TLR5, and TLR9 localization in gastric premalignant lesions before and after Helicobacter pylori eradication. Villarroel-Espíndola et al. developed a multiplex immunohistochemistry/immunofluorescence (mIHC/IF) technology for the simultaneous detection of TLR4, TLR5, and TLR9 on a single tissue section of human gastric biopsies. Utilizing paired tissues collected over a longitudinal three-year interval from patients undergoing Helicobacter pylori eradication therapy, the study traced receptor density across chronic atrophic gastritis and intestinal metaplasia. Spatial cartography demonstrated that while both cell-surface epithelial TLR4 and endosomal TLR9 are upregulated by active H. pylori presence, only TLR9 correlates directly with the severity of intestinal metaplasia. In contrast, TLR5 is strictly confined to infiltrating stromal mononuclear cells and specialized goblet cells, showing no association with active bacterial loads. This spatial tracking offers a promising methodology to refine individual gastric cancer risk assessment when clinical antibiotic eradication fails [59].

In another study, Pallua and Schirmer characterized immune and bone biomarker trajectories within a proteoglycan-induced murine spondyloarthritis (SpA) model. By comparing Th1-prone C57BL/6J and Th2-prone BALB/c backgrounds alongside immunized TLR2, TLR3, and TLR4 knockout models, the authors perform a longitudinal assessment of 26 circulating cytokines paired with terminal bone turnover markers. Their findings demonstrate model-specific systemic polarization. Proteoglycan immunization triggered a robust pathogenic Th1/Th17 axis (TNF-α, IFNγ, IL-12p70, IL-17A, and IL-22) within the C57BL/6J background, mirroring human axial SpA parameters. In contrast, immunized BALB/c mice exhibited a marked type 2 eosinophil-associated profile characterized by high IL-4, IL-5, IL-9, and IL-13. Within the C57BL/6 background, genetic deletion of either TLR2 or TLR3 significantly reduced Th1 and Th17 cytokine cascades while shifting systemic balance toward type 2 mediators, validating that innate pattern recognition receptors are vital to sustain pathogenic joint inflammation. Furthermore, TLR2-deficient mice present significantly elevated terminal serum levels of Dickkopf-1 (DKK1) and Noggin, indicating a functional link between innate immune sensing and systemic bone remodeling pathways [60].

Bridging metabolic state with systemic immune reactivity, the next study investigated the capacity of dietary omega-3 (n-3) polyunsaturated fatty acids (PUFAs) to reshape lipid mediator profiles within retroperitoneal white adipose tissue (WAT). Wenderoth et al. paired genetic enrichment models (transgenic fat-1 mice) with strict omega-3 sufficient or deficient diets to track specialized pro-resolving lipid mediator (SPM) kinetics in retroperitoneal white adipose tissue (WAT) during lipopolysaccharide-induced endotoxemia. High-resolution liquid chromatography–tandem mass spectrometry (LC-MS/MS) proved that nutritional and genetic omega-3 enrichment significantly drives downstream conversion pathways. Under severe LPS-induced challenge, enriched WAT markedly increased the production of resolution-phase oxylipins while actively suppressing the arachidonic acid cascade. Concurrently, the omega-3 sufficient background prevented the systemic transcription collapse of key protective adipokines like adiponectin and leptin. This dual metabolic–lipidomic response establishes that WAT functions as a major functional repository releasing circulating oxylipins to mitigate systemic inflammatory shocks [61].

The last study of this collection addresses an alternative, non-competitive mechanism of small-molecule receptor stabilization that holds promise as a template for pharmaceutical design. Del Santos et al. characterized Mygalin—a low-molecular-weight (417 Da) synthetic acylpolyamine modeled after a natural compound isolated from the hemocytes of the spider Acanthoscurria gomesiana—as an antagonist of the TLR2 pathway. In macrophage lines stimulated with diverse TLR2/1 and TLR2/6 ligands (Pam3CSK4, Pam2CSK4, and Zymosan), Mygalin pre-treatment strongly suppressed nitric oxide (NO) synthesis and IL-6 production by downregulating NF-κB p65 and STAT-1 phosphorylation. In silico models demonstrated that Mygalin does not fit into the deep hydrophobic pockets of TLR1, TLR2, or TLR6. Instead, it binds selectively to the solvent-exposed neck boundary linking the monomers, functioning as a structural wedge that stabilizes the inactive resting-state heterodimer to block the conformational rearrangements required for intracellular signaling [62].

## 2. Discussion

Building on the previous two volumes [39,63] of this Special Issue series, the present collection further illustrates the dual role of TLRs as drivers for the pathogenesis of acute bacterial and viral infections as well as non-infectious inflammations but also as potent players for antiviral, antibacterial, and anti-cancer immune activation. The articles presented in the present Special Issue provide an interconnected collection of structural, cellular, pre-clinical, and clinical insights that enrich our understanding of TLR biology, ranging from structural alignments in receptor binding to translational interventions further to clinical applications. For instance, in silico analyses provide a unifying hypothesis [53] on how highly conserved hydrophobic coiled-coil motifs within viral fusion core structures broadly shared by enveloped viruses can stabilize human TLR4/MD-2 dimerization, providing a possible molecular explanation for the acute Th1-driven pulmonary destruction frequently observed during acute viral infections [36,37,38,39,40,41,42,43,44,45,46,47,48,49,50,51,52,53], including influenza virus infection, where tissue damage correlates with a TLR-dependent cytokine storm [54]. 

The elucidation of these pathophysiological cascades provides a rationale for therapeutic modulation of TLR-recognition and signaling such as described in the work of Zhang et al., whose development of the intracellular TLR4 inhibitor DB03476 supports that structural blockade of the intracellular TIR pocket can rescue downstream MyD88-mediated cytokine storm [57]. TLR modulation can be expanded also to alternative approaches, such as via Mygalin-mediated locking of the TLR2 heterodimer neck [62].

Another thematic bridge connects intracellular nucleic acid sensing with altered extracellular TLR responses. Di Rosa et al. show that cGAS-STING activation primes the expression of BCAP, which then alters the host cell response to a subsequent extracellular LPS-TLR4 challenge, steering it toward type I interferon synthesis while actively suppressing pro-inflammatory TNF-α [60]. This cellular rewiring aligns with the findings of Wenderoth et al., who describe how altering the tissue microenvironment—in this case, via white adipose tissue enrichment with omega-3 PUFAs—can reset host responsiveness to systemic LPS endotoxemia, switching the tissue profile from pro-inflammatory eicosanoids to resolution-promoting SPMs [61].

Furthermore, the balance between acute inflammatory tissue destruction and late-stage immunomodulation is reflected in the genetic studies of Flores-González et al., who show that non-internalizing TLR2 synonymous variants promote aberrant cytokine and regulatory IL-10 profiles (Flores-González et al.). It also parallels the work of Pallua and Schirmer, who show that genetic deletion of TLR2 or TLR3 alters systemic Th1/Th17 polarization and profoundly shapes terminal bone marker kinetics [60].

Finally, the intentional activation of these different loops can be utilized for therapeutic benefit, as demonstrated by Dronamraju et al., who employ prophylactic FSL-1 administration to stimulate the TLR2/6/MyD88 matrix, turning a potentially destructive inflammatory cascade into a survival signal that accelerates hematopoietic recovery [56]. 

Collectively, these articles underscore that Toll-like receptors are not static, isolated switches. Instead, they function as dynamic, highly tunable molecular networks whose spatial orientation, genetic background, and microenvironmental and temporal context dictate the fine line between protective immunity and severe inflammatory disease [64,65,66,67,68,69,70,71,72,73,74,75,76,77].

## 3. Conclusions

TLRs contribute to the pathogenesis of both acute bacterial and viral infections as well as a broad range of non-infectious inflammatory conditions. At the same time, however, they serve as essential environmental sensors and triggers for antiviral, antibacterial, and anti-cancer surveillance. The insights gathered within this collection together with the previous volumes of this Special Issue series highlight the broad range of implications and applications of the TLR field while highlighting crucial priorities for future research. The successful integration of high-resolution modeling with in vitro organoids and in vivo models suggest that structural biochemistry can predict and shape macro-organismal pathology and propose modalities for treatment. The next frontier in TLR therapeutics lies in exploiting this structural resolution, i.e., by transitioning from broad, systemic receptor blockade—which risks compromising critical host immune surveillance—to target-specific, conformation-sensitive modulators, by which it will become possible to achieve therapeutic precision.

As demonstrated by the diverse inputs in this collection, therapeutic success will depend on temporal and contextual accuracy: utilizing small-molecule antagonists or natural inhibitors to lock hyper-activated receptors during acute hyperinflammation, while strategically deploying specific agonists to prime hematopoietic or antiviral, antibacterial, and anti-cancer resilience. Ultimately, unraveling the complex crosstalk between lipid microenvironments, genetic variations, and downstream adaptor loops will hopefully empower scientists and clinicians to translate this knowledge into improved strategies for controlling and managing host immune response across infectious and non-infectious inflammatory pathologies and metabolic diseases.
